# Bench to Bedside: Animal Models of Radiation Induced Musculoskeletal Toxicity

**DOI:** 10.3390/cancers12020427

**Published:** 2020-02-12

**Authors:** Michael K. Farris, Corbin A. Helis, Ryan T. Hughes, Michael C. LeCompte, Alexander M. Borg, Karina Nieto, Michael T. Munley, Jeffrey S. Willey

**Affiliations:** Department of Radiation Oncology, Wake Forest School of Medicine, Winston-Salem, NC 27157, USA; mfarris@wakehealth.edu (M.K.F.); chelis@wakehealth.edu (C.A.H.); ryhughes@wakehealth.edu (R.T.H.); mlecompt@wakehealth.edu (M.C.L.); aborg@wakehealth.edu (A.M.B.); knieto@wakehealth.edu (K.N.); mmunley@wakehealth.edu (M.T.M.)

**Keywords:** radiation therapy, fracture, normal bone toxicity, osteoclast, osteoblast, SBRT, cortical thickness mapping

## Abstract

Ionizing radiation is a critical aspect of current cancer therapy. While classically mature bone was thought to be relatively radio-resistant, more recent data have shown this to not be the case. Radiation therapy (RT)-induced bone loss leading to fracture is a source of substantial morbidity. The mechanisms of RT likely involve multiple pathways, including changes in angiogenesis and bone vasculature, osteoblast damage/suppression, and increased osteoclast activity. The majority of bone loss appears to occur rapidly after exposure to ionizing RT, with significant changes in cortical thickness being detectable on computed tomography (CT) within three to four months. Additionally, there is a dose–response relationship. Cortical thinning is especially notable in areas of bone that receive >40 gray (Gy). Methods to mitigate toxicity due to RT-induced bone loss is an area of active investigation. There is an accruing clinical trial investigating the use of risderonate, a bisphosphonate, to prevent rib bone loss in patients undergoing lung stereotactic body radiation therapy (SBRT). Additionally, several other promising therapeutic/preventative approaches are being explored in preclinical studies, including parathyroid hormone (PTH), amifostine, and mechanical loading of irradiated bones.

## 1. Introduction

Approximately half of the 1.7 million new annual cancer diagnoses will be treated with ionizing radiation therapy (RT). As improvements in cancer screening and treatments continue to decrease cancer mortality, patients are now living long enough to experience treatment-related toxicities that may have previously been obscured by cancer-related death. Mature bone has classically been thought of as radio-resistant tissue; however, recent pre-clinical and clinical studies have demonstrated that RT-induced bone loss and fracture are common problems with serious implications for patient quality of life (QOL) [1,2,3].

Stereotactic body radiation therapy (SBRT), for example, when used in the treatment of lung tumors near the chest wall, unavoidably delivers high doses of radiation to the ribs or vertebrae, and it can predispose patients to significant risks of radiation-induced rib fractures (RIRF), vertebral fractures, and chest wall pain (CWP) [4,5,6]. These toxicities have been correlated with the dose received by the chest wall. Approximately 35% of patients develop grade 3 or higher CWP requiring medical intervention with anti-inflammatory drugs or opioids when greater than 30 cubic centimeters of chest wall receives 30 gray (Gy) or more [7]. The incidence of RIRF within the first year of SBRT has been reported to be as high as 40% [5,8,9,10,11,12,13]. Patients can experience severe pain, compromised ventilation, and impaired QOL. In the elderly, decreased oxygen saturation as a result of compromised ventilation can reduce life expectancy [14].

As SBRT becomes a new standard approach for treating tumors throughout the body in the oligometastatic setting, the incidence of RT-induced bone toxicity is forecasted to increase over time [15,16,17,18,19]. While dose–response relationships have been described and will be reviewed in detail below, it is important to note that RT-induced bone injury is not limited to just ribs and vertebrae or high doses of RT per fraction.

In women treated for endometrial adenocarcinoma with standard doses of RT per fraction (1.8–2 Gy per fraction), the 5-year pelvic fracture rate is ~30% [20,21]. Likewise, hazard ratios for RT-induced hip and femoral neck fractures indicate significant increased risk at five years post-RT for cervical (1.66), rectal (1.65), and anal (3.16) cancers [22]. Alarmingly, the rate of incomplete healing is high with RT-induced fractures, which greatly contributes to morbidity [23,24]. Therefore, RT-induced bone damage is a persistent and major source of functional impairment and pain in many cancer survivors.

The exact mechanisms of RT-induced bone damage are unclear. Utilizing clinical data alone to determine the effects of RT on bone is complicated by numerous confounding factors, including medical comorbidities such as osteoporosis/osteopenia or concurrent medications that may impact bone turnover/structure, such as chemotherapy or steroids. Therefore, research with animal models has been critical in identifying several mechanisms by which RT-induced bone injury may occur, as well as in identifying potential therapeutic approaches. Below we will: (i) review the preclinical studies that helped elucidate the relationship between irradiation and bone injury, (ii) focus on how these data have been applied to develop approaches for addressing clinical research questions related to radiation-induced bone loss, and finally, (iii) review how the most recent rodent studies have helped generate multiple therapeutic approaches.

## 2. Results and Discussion

### 2.1. Mechanisms of Radiation-Induced Bone Changes

Numerous studies have demonstrated that skeletally mature humans who are exposed to RT experience higher incidence of bone fractures, decreased bone strength, and osteoporosis [9,20]. The exact mechanisms of RT-induced bone damage have not been fully elucidated. Nutritional status, activity levels, weight bearing, chemotherapy, steroid use, and underlying comorbidities all play a significant role in the manipulation of the complex physiologic balancing act that is bone turnover [25,26,27,28,29]. Skeletal maturity also has an impact on the sensitivity to irradiation. Immature bone has a higher turnover than mature bone, and it is therefore more sensitive to perturbations in bone hemostasis [30]. Pediatric cancer survivors who were irradiated prior to skeletal maturity also demonstrate growth retardation, scoliosis, and increased incidence of fractures later in life [30,31,32]. 

These confounding factors often cloud the true impact of irradiation, but most of these factors are difficult to control in a clinical setting. Human data on irradiation of normal bone without the impact of weight bearing is mostly possible through research involving low earth orbit astronauts exposed to cosmic irradiation. Controlled preclinical studies however, utilizing murine and nonhuman primate models, have greatly advanced our understanding of the effects that irradiation has on the bone microenvironment both before and after skeletal maturity. As we gain more insight into the precise mechanisms of damage, we can begin to formulate preventative strategies for those at the highest risk of meaningful bone toxicities.

Historically, the mechanisms of RT-induced bone damage were thought to be related to vascular injury as well as persistent osteoblast damage/suppression [33,34]. Damage to osteoblasts and progenitors has long been considered as a primary contributor to the development of reduced bone mineral density [33,35,36,37,38,39,40]. Several studies examining radiation-toxicity in normal, non-cancerous bone have focused on osteogenic and colony-forming potential of mesenchymal stem cells post-RT [41,42]. Lower numbers of osteoblasts are observed within irradiated bone [36,37,38,43]. Moreover, an increase in reactive oxygen species and associated damage to DNA [44] and related apoptosis is a putative mechanism for cell death [37,39,45,46]. Overall, this effect on osteoblasts and progenitors may lower new matrix (e.g., collagen) production and lead to a reduction in bone density that can increase the risk of fracture [36,47,48,49,50,51]. As such, protecting the osteoblast is a therapeutic strategy for maintaining bone health after radiation therapy.

An acute, transient increase in the number and activity of osteoclasts is a putative mechanism for causing the pathologic fractures in bone that are caused by radiation therapy [49]. Therefore, suppressing radiation-induced osteoclast activity represents a potential and realistic therapeutic approach for preventing radiation-induced fractures given that the Federal Drug Administration (FDA)-approved osteoporosis medications frequently and effectively suppress osteoclast activity (e.g., bisphosphonates). Multiple investigators have reported increased osteoclast number and activity post-exposure [43,46,49,52,53,54], that can lead to early bone loss [54]. Oest et al. investigated osteoclast numbers and mineral apposition rate (MAR) over time after focally irradiating the hind-limbs of BALBc/J mice with 20 Gy in five fractions [55]. Histologic samples were analyzed at 1, 2, 4, 8, 12, and 26 weeks after RT, and it was found that early osteoclast numbers were transiently increased by week 2 but then at later time points, a persistent osteoclast depletion was observed. The transient increase was most prevalent among trabecular osteoclasts and was felt to contribute to trabecular resorption that was not later restored. The persistent depletion of osteoclasts permitted unopposed cortical thickening through imbalanced bone remodeling. The resulting bone was more fragile due to the accumulation of micro-damage and matrix embrittlement. 

The cause for an early and transient increase in osteoclast activity is unclear, and perhaps due to signals between other cells, including osteocytes. Bone is made up of approximately 90–95% osteocytes [56]. These cells serve as the conductors of homeostasis. While osteocytes are buried in the matrix of lacunae, their dendritic processes allow sensory information from bone stressors (including radiation) to trigger the release of inhibitory or stimulatory signals, which drive the response of osteoclasts, osteoblasts, and osteoprogenitor cells. A link exists between osteocyte dendritic morphology and apoptosis from radiation and subsequent differentiation of osteoclasts [37,56,57], perhaps through expression of receptor activator of nuclear factor kappa B-ligand RANKL, lowered osteoprotegerin (OPG), and high-mobility group box 1 protein (HMGB-1) identified from the irradiated osteocyte cell line MLO-Y4. 

**Figure 1:**
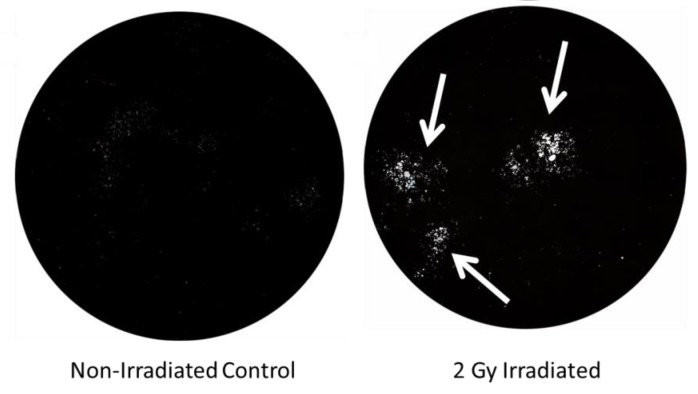
2 Gy increases osteoclast activity, resulting in a 390% increase (*p* < 0.05) in resorbed surface area of hydroxyapatite (white spots indicated by arrows). *n* = 3 replicates.

This increase in osteoclast differentiation can be demonstrated by isolating marrow from irradiated bone marrow and cultured on a proxy bone surface. To further investigate this finding and directly measure osteoclast activity, hematopoietic cells from the femurs of C57BL/6 mice can be isolated, and then plated on a hydroxyapatite (HA) proxy for bone in 12-well plates using RANKL and macrophage colony-stimulating factor (MCSF) to promote osteoclast differentiation. By fourteen days after a 2 Gy X-ray dose, erosion of the HA surface area is increased by 390% relative to control (Figure 1). 

Overall, this acute increase in osteoclast activity leads to the hypothesis that osteoclast inhibition utilizing a bisphosphonate (e.g., risedronate) could prevent or reduce RT-induced bone loss and prevent fractures [58]. In 2010, this hypothesis was explored with a group of 115 skeletally mature (20-week-old) C57BL/6 mice [38]. Mice were split into groups of no RT + placebo, RT + placebo, or RT + risedronate 30 μg/kg every other day. The irradiated mice were treated with 2 Gy X-rays to the whole body. Mice were sacrificed at 1, 2, and 3 weeks following RT, and the proximal tibial metaphysis, distal femoral metaphysis, and lumbar vertebrae were assessed with micro-computed tomography (CT). In irradiated animals receiving placebo, micro-CT at all locations demonstrated a reduction in trabecular volume ratio, as well as loss of connectivity and trabecular number when compared to non-irradiated mice. The majority of bone loss had occurred during the first week following RT. By week 2, there was decreased trabecular and endocortical bone formation. As expected, tartrate-resistant acid phosphatase (TRAP) 5b, a biomarker for osteoclastic bone resorption that is released from blood and can be quantified from serum, was increased by 1 week in the irradiated mice given placebo compared to non-irradiated mice. Indeed, the efficacy of bisphosphonates (zoledronate) was shown in protecting radiation-induced bone loss in the femurs of mice exposed to a high dose fraction (20 Gy) delivered to the hind limb [59], and reducing biomarkers of bone resorption measured from ^45^Ca kinetics late after exposing BALB/c mice to a single 16 Gy fraction that delivered a clinically-relevant biologically effective dose (BED) of 101.3 Gy [60]. However, it should be noted that administration of bisphosphonates has not been efficacious in all rodent models [39]. Rats treated with a clinically relevant schedule of 8 × 2 Gy fractions delivered to the hind limbs exhibited lower bone volume fraction within the irradiated HL by 4 weeks post exposure without sparing with alendronate administration, though dose and delivery were not identified. 

### 2.2. Development of a Method for Measuring RT-Induced Bone Toxicity

Several of the above-noted rodent studies utilized primarily histologic stains to demonstrate changes in osteoclasts, as well as trabecular number, density, and cortical thickness. Micro-CT also improves upon the evaluation of trabecular volume/number and cortical thickness, but is limited to small regions of interest. A novel three-dimensional (3D) cortical mapping algorithm was developed that could easily be scalable to larger CT scans, multiple bones simultaneously, and provide both quantitative and qualitative information on RT-induced bone changes [4,61,62]. 

In the creation of this algorithm, the entire vertebral column of 16 rhesus macaques nonhuman primates (NHPs) were assessed before and after these animals had received whole chest irradiation to 10 Gy to the midplane in a single fraction, delivered with 6-MV X-rays in an Anterior-Posterior / Posterior-Anterior (AP/PA) fashion at a dose rate of 6 Gy/min [61]. All NHPs had full body CT scans at 2 months prior to RT, then post RT at 2, 4, 6, and 8 months. 

For each CT scan, the bony anatomy was segmented using automated thresholding techniques and manual editing in Mimics (v16.0 Materialize, Leuven, Belgium) for Windows. Individual contours of each vertebral body were constructed in treatment planning software and these contours were then utilized to construct 3D surfaces containing over 2000 orthogonal vertices using Stradwin software (v5.0 Cambridge University, Cambridge, England) for Windows. The global cortical bone density for each CT scan was used and collected density measurements at each vertex. 

For both skeletally mature and immature animals, the resulting cortical thickness maps showed a significant infield cortical thickness loss at the first imaging time point, 2 months post RT [61]. After the initial infield cortical thickness decrease, the effect did not continue to decrease, but rather remained persistently lowered from baseline until the final CT scan at 8 months post RT. This implied that the bulk of RT-induced damage occurred quickly and was subsequently not repaired. The cortical thickness did not decrease significantly outside the radiation portal. The rapid drop in cortical thickness was pronounced in skeletally immature NHPs, which was expected given their higher baseline bone turnover rate and hence, increased RT sensitivity. Finally, when paired with histologic assessment of vertebrae, our cortical thickness maps were closely correlated with the thinning of cortical bone observed on H&E-stained bone sections. 

This study demonstrated that in-field RT-induced bone changes occur rapidly, and are unlikely to be repaired. Additionally, it illustrated that our mapping approach was truly reflective of *in vivo* changes given the close correlation of our findings with histologic photomicrographs. 

This same cortical thickness mapping approach was applied to a retrospective database of lung cancer patients with peripheral lung tumors receiving SBRT to estimate changes in rib cortical thickness. Patients were treated to 50 Gy at 5 to 10 Gy per fraction. Baseline CT planning scans were compared to 3-month post-SBRT follow-up scans. Using iterative closest point (ICP) registration in MATLAB (v2014a, MathWorks, Natick, MA), one can register received radiation doses to the surface of each rib to the corresponding cortical thickness map [4]. 

By 3 months post SBRT, we were able to detect rapid cortical thickness loss in field. Furthermore, we were able to describe a continuous dose–response relationship. Regions of ribs that received 10 Gy or more exhibited significant cortical thickness loss at ~7%, with a range of 4% to 11%, but with every additional 10 Gy interval, the degree of cortical thickness loss increased. Regions receiving 40 Gy or more had the greatest cortical thickness loss at 18%, with a range of 15–22% loss. In areas of the ribs receiving >30 Gy, approximately 92% of treated patients developed significant cortical thinning by 3 months, with RIRF occurring as early as 6 months post RT [4].

Utilizing this mapping approach with lower doses per fraction, cortical thinning was once more correlated with total dose received by bone. The CT scans from 23 anal cancer patients receiving 53–58 Gy at 1.8 Gy per fraction were retrospectively analyzed at baseline and <4 months post RT. Again, significant early cortical thinning (< 4 mo) was noted in the intertrochanteric crest, and femoral neck. The femoral neck volume of bone receiving 40 Gy was a significant predictor of focal cortical thinning of 30% or more [62]. 

### 2.3. Application of Cortical Thickness Mapping for Osteoradionecrosis (ORN) of the Jaw

ORN of the mandible after head and neck RT is a significant issue affecting cancer patients who receive RT as part of their curative treatment [63]. Over 60,000 head and neck cancer patients are diagnosed each year, many of whom are treated with RT. These patients are at high risk of RT-induced bone toxicity given the close proximity of the mandible to the RT target. ORN of the mandible is a feared complication of RT and represents non-healing, non-vital bone within the RT field. It occurs in 5–38% of patients and may present with serious symptoms, including exposed bone, pathologic fracture, intra- and extra-oral fistula, and infection. It is associated with factors including dental extractions (both pre- and post-RT), surgical intervention, tobacco use, diabetes, and RT dose [64,65,66,67]. The treatment of ORN is largely ineffective and consists of conservative management (antibiotics, oral hygiene, and pain medication), anti-fibrotic agents (pentoxyfylline), hyperbaric oxygen, and surgical debridement [68,69,70]. ORN is a significant clinical issue for which there has been little innovation to date. Given improvements in survival, particularly for those with human papilloma virus (HPV)-associated oropharyngeal cancer, RT effects on the mandible remain a critical issue that has been without biomarkers or molecular targets for as long as RT has been used in the head and neck. Methods to quantify early mandible bone loss are needed to inform further study to minimize the risk of ORN after head and neck RT.

**Figure 2:**
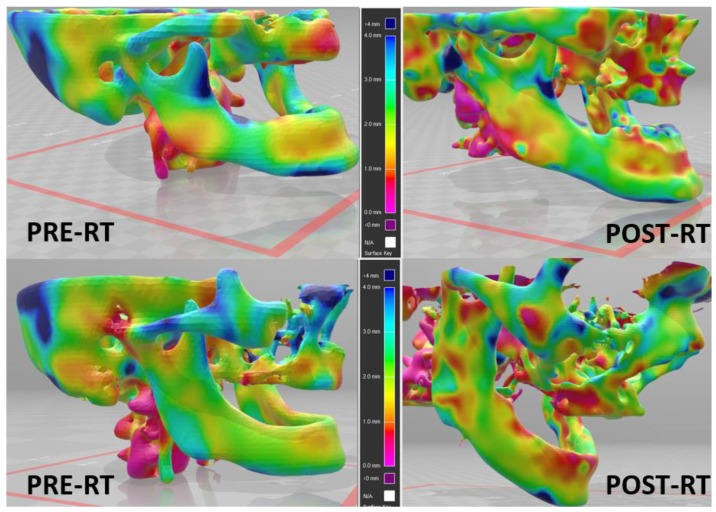
Early cortical bone thickness loss after radiation therapy (RT) for two patients with oropharyngeal squamous cell carcinoma treated with chemo-radiotherapy. Left: pre-RT, right: post-RT (median 90 days after end of RT).

The pathogenesis of mandible ORN is thought to be secondary to a combination of tissue hypoxia, hypocellularity, and vascular changes [71]. While this may be the case for the chronic phase of the disease [72], early bone changes in the mandible that may predispose to ORN have not been well characterized. Further study of this process is difficult to translate to the clinic without the ability to quantify early mandible cortical bone changes. Our cortical mapping methodology described above, which was initially developed in NHP models, has also been applied to the mandible in patients receiving head and neck RT (Figure 2). Quantification of early mandible bone loss that correlates with ORN would identify an intervenable mechanism for subsequent clinical trials aimed to minimize the risk of ORN. 

### 2.4. Antiresorptive (Bisphosphonate) Strategy for Prevention of RT-Induced Bone Toxicity

**Figure 3:**
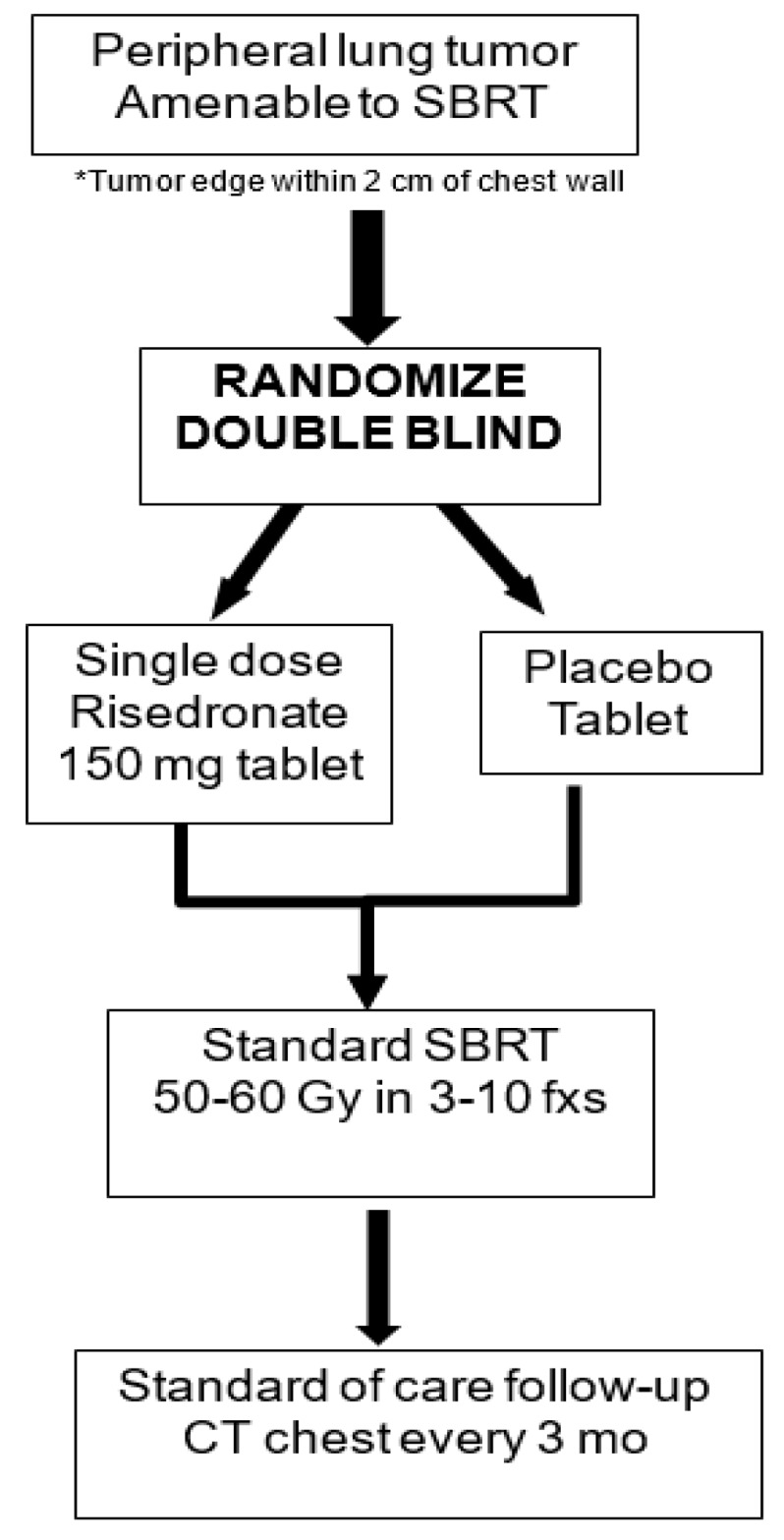
Study Schema. SBRT, stereotactic body radiotherapy. Placebo = methylcellulose; Study drug to be given approximately 10 days prior to SBRT start.

Based on these preclinical data utilizing mouse and NHP models, as well as retrospective clinical studies, a clinical protocol to assess and potentially prevent SBRT-induced bone loss has been developed and activated. For this ongoing trial, the aim is to determine if single dose prophylactic suppression of early RT-induced osteoclast activity with bisphosphonates diminishes or prevents RT-induced cortical bone thinning, fracture, and pain. As demonstrated in Figure 3, a total of 84 patients with peripheral lung tumors (within 2 cm of the chest wall) will be randomized in a double-blind fashion to either a single dose of risedronate versus placebo, given at least 7 days prior to initiation of SBRT. All patients will be treated with standard of care SBRT of 50–60 Gy in 3 to 10 fractions in accordance with our institutional practice. In the setting of peripheral tumors, it is particularly challenging (if at all possible) to meet commonly published normal tissue constraints of the rib/chest wall. While clinicians are encouraged to attempt to limit the rib/chest wall dose as much as possible, there is no maximum dose limit placed on these structures. The study has been granted an FDA Investigational New Drug (IND) exemption (IND # 142767), is Internal Review Board (IRB) approved, and currently enrolling patients NCT03861091.

The primary objective is to assess the percent change in bone mean cortical thickness within regions receiving 30 Gy or more at 3 months post SBRT. Secondary endpoints will also assess cortical thickness change at 6, 9 and 12 months after SBRT. The percent change in mean cortical thickness will be compared between patients who received risedronate and those who received placebo. Mean cortical thickness will be measured for the ribs and vertebrae within the irradiated field using our cortical thickness and radiation dose mapping approach. The incidence of RIRF within the treatment field will be recorded. CWP at each time point will also be assessed utilizing a modified Radiation Therapy Oncology Group (RTOG) grading scale. 

As a biomarker correlate, urinary metabolites of bone breakdown will be assessed at each time point. A variety of urinary biomarkers have been linked to increased osteoclastic activity in osteoporotic populations, and the most frequently tested marker is urinary N-terminal telopeptide crosslinks (NTX) [73]. These biomarkers have been assessed following RT to the pelvis with or without bisphosponates and were shown to decrease with the addition of bisphosphonates [58]. In the nonhuman primate chest irradiation study we also detected elevated urinary NTX supporting irradiation-induced upregulation of osteoclasts. 

Bisphosphonates are generally well tolerated, however there is a low risk of osteoradionecrosis (ORN) of the mandible. Amongst patients on chronic risedronate therapy, there was a ~1–12% [74] risk of ORN of the mandible. Given that this study will provide a single dose of risedronate, we anticipate that this risk will be further reduced. 

There is no reported increased incidence of toxicity in patients receiving chest RT. Concurrent bisphosphonates are not contraindicated prior to chest SBRT. Importantly, none of the patients in this study are receiving head and neck irradiation. Similarly, patients with dental operations involving manipulation of bone within the last 6 months are excluded. 

**Figure 4:**
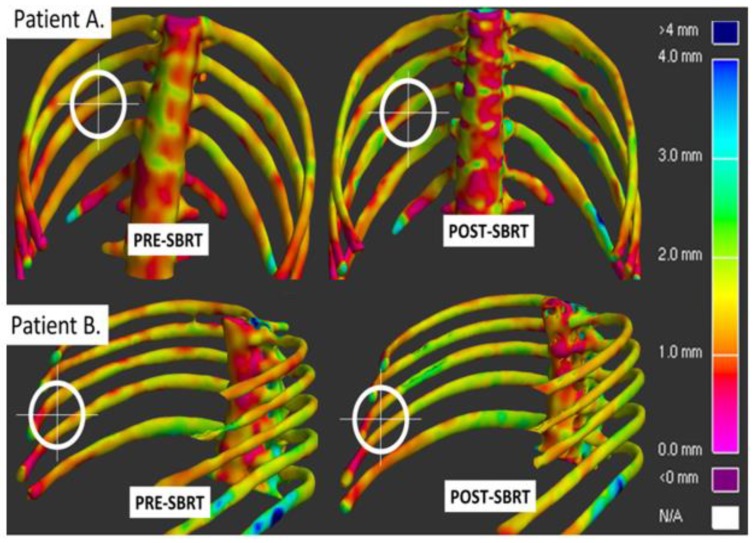
Minimal cortical thinning was observed in the ribs of two lung cancer patients who were taking risedronate for osteoporosis at the time of SBRT. The tumors were located near the rib wall. The rib regions of interest are within the white “target” which is adjacent to the tumor and received >30 Gy. The lack of thinning is noted by no marked increase in red coloration of bone in the post SBRT scans.

Additionally, our group performed an initial retrospective analysis of a very small number of patients, *n* = 2, with peripheral lung tumors who were incidentally taking bisphosphonates when they were treated with lung SBRT. We found no increased risk of any toxicity from concurrent SBRT and bisphosphonate therapy. Furthermore, on cortical thickness mapping, these patients had minimal cortical thinning of the ribs near the irradiated site (Figure 4). 

In summary, this prospective randomized study will be the first clinical trial to investigate whether suppression of osteoclastic activity in patients prevents or ameliorates irradiation-induced early rib and vertebral bone loss and late rib and vertebral fractures in regions receiving >30 Gy. Additionally, it will help to parse out the impact that bone breakdown has on development of RT-induced CWP. Finally, it will potentially provide a simple and safe prophylactic measure for patients at the highest risk of RT-induced bone toxicity. 

### 2.5. Alternative Strategies for Prevention of RT-Induced Bone Toxicity

While osteoclast inhibition may represent a promising clinical target for bone radioprotection, bisphosphonates may be contraindicated. For example, they may be contraindicated due to renal dysfunction, or history of allergy to bisphosphonates. Additionally, given the increased risk of ORN of the mandible, bisphosphonates should be avoided in patients receiving head and neck RT (which also portends an increased risk of ORN).

For this reason, several alternative strategies for the prevention of RT-induced bone toxicity have also been explored. These include the use of parathyroid hormone (PTH), sclerostin antibody, amifostine, angiogenesis manipulation, anabolic weight loading, and antioxidant dietary supplementation. 

#### 2.5.1. PTH and Blocking Sclerostin 

As noted, anabolic approaches are strong candidates for the prevention of RT-induced fractures due to the known effects on osteoblasts [37]. Based on its use in severely osteoporotic patients, intermittent administration of parathyroid hormone may be a useful drug to decrease bone loss and reduce the risk of fracture. The use of PTH has been investigated in a series of studies by Oest and colleagues [51,75]. In the first, they investigated the impact of PTH on bone fragility in a mouse hind limb model. All mice received 20 Gy in four fractions to a hind limb with the contralateral limb as a control, with either subcutaneous PTH or placebo for eight weeks starting on the day of the first fraction of RT. They noted improvements in loss of trabecular bone, cortical bone volume, and mechanical strength of bone with the addition of PTH. However, these benefits were rapidly lost after PTH was stopped. In the second study (using the same murine model), they noted an increase in pathologic collagen crosslink in radiated bones, as well as changes in the mineral crystallinity and mineral-to-matrix ratio. Treatment with PTH partially improved the collagen crosslink ratio, but did not restore changes in collagen or mineral alignment. The long-lasting changes in the matrix caused by RT and not impacted by PTH may explain the rapid increase in fragility once PTH was withdrawn in the first study. Moreover, blocking sclerostin, which promotes bone formation through effects on the Wnt signaling pathway, has also been shown to be efficacious [37]. 

Kang and colleagues also investigated the use of PTH to mitigate the effects of RT on bone using a murine model of distraction osteogenesis [76]. Mice were exposed to 35 Gy of RT and treated with intermittent recombinant PTH or a placebo. A substantial decrease in the number of mandibles exhibiting a complete union was seen in subjects receiving RT compared with the controls (43% versus 100%). However, the addition of PTH increased the union rates to 80%. They additionally showed that intermittent PTH restored the osteocyte count of the irradiated bone to that of the control group. Mature bone formation was also improved with the addition of intermittent PTH, although not enough to restore it to the levels of the control non-irradiated group.

While preliminary and published data on PTH is promising, there is also a concern (albeit controversial) that PTH may induce osteosarcomas. This has been shown in limited animal models and case reports in humans with primary hyperparathyroidism leading to prolonged PTH elevation [77,78,79]. This has led to an FDA warning for the use of PTH, and could limit its use for the prevention of RT-induced fractures should this concern not be reversed.

#### 2.5.2. Amifostine

Amifostine is a radio-protective agent that acts by scavenging free radicals generated by ionizing radiation. A recent *in vitro* study of rat bone marrow stem cells with or without radiation and amifostine showed that the addition of amisfostine to irradiated cells decreased the amount of reactive oxygen species and DNA damage and increased the levels of cellular proliferation, which is promising [80]. Additionally, Zhang and colleagues showed that in an *in vivo* rat model, by reducing DNA damage, amifostine may decrease the differentiation of osteoclast precursor cells into differentiated osteoclasts. Furthermore, they noted decreased lacunar resorption pits in rodents treated with RT and amifostine than those with RT alone [81]. 

These findings show promise that amifostine may be able to reduce RT-induced bone damage. However, there are substantial challenges to this strategy that need to be overcome for routine clinical use for this indication. First, amifostine must be administered intravenously shortly before the delivery of RT, which presents logistical challenges. Furthermore, substantial side effects have been reported in human clinical trials of amifostine, including a Phase III trial of amifostine that was terminated early due to severe toxicity in 41% of patients. A review of the literature in the report of the terminated trial noted an overall 27% rate of amifostine discontinuation due to severe adverse events [82]. However, as noted, amifostine use in rodent models shows promise as a therapeutic agent. 

#### 2.5.3. Angiogenesis Manipulation

Angiogenesis is a critical component of bone modeling and remodeling. Tong and colleagues hypothesized that the effects of RT on bone marrow-derived endothelial progenitor cells may also impact the resorption of bone after RT. In an *in vitro* study, they found that RT induced substantial dysfunction in endothelial progenitor cells, including the downregulation of vascular endothelial growth factor A (VEGF-A), which is a potent angiogenic agent [83]. However, angiogenesis is a critical part of tumor growth and metastasis, and abnormally upregulated angiogenesis, specifically VEGF, is a direct target of many commonly used systemic therapy agents, including bevacizumab [84]. This makes attempts to target downregulated angiogensis as a method to prevent RT-induced bone toxicity in cancer patients be of questionable value. 

#### 2.5.4. Anabolic Weight Loading

Sufficient mechanical loading is critical for proper bone development, leading to increased bone formation and decreased bone loss [85]. However, whether this can be used to mitigate the effects of RT on bone has not been well studied. A recent study by Govey and colleagues used a murine bone marrow transplant model to investigate the use of mechanical loading on bone loss [86]. They found that irradiated tibias that underwent mechanical loading lost 31% less trabecular bone volume and 8% less cortical thickness than irradiated tibias without mechanical loading. Current NCCN clinical guidelines for cancer survivorship recommend at least 150–300 min of moderate intensity of 75 min of vigorous intensity per week, including two to three sessions of strength training per week [87]. Additional emphasis on this in patients at risk of RT-induced bone toxicity may be an easily implementable method of decreasing clinical toxicity. Care must be considered for all affected skeletal locations, and many patients are not able to undertake sufficient exercise due to pre-existing medical conditions or other effects of therapy. Additionally, care must be given to not overload the bones in question, which may lead to pathologic fractures

#### 2.5.5. Dried Plum Diet

Finally, dried plums are rich in antioxidants, and have been shown to prevent bone loss in murine osteoporosis models. However, until recently, it was not as well studied for RT-induced bone loss. In a murine model, Schreurs et al. showed that dietary supplementation with dried plums decreased the expression of multiple genes involved in bone resorption, including RANKL and TNF-a [88]. Additionally, the dried plum-supplemented diet did not have any cancellous bone loss after 2 Gy radiation exposure, while those fed the control diet had substantial bone loss. These preclinical results are promising; the dried plum-supplemented diet contained 25% dried plums by weight, which may provide some challenges to address for use in routine clinical practice.

## 3. Conclusions

In summary, bone is a radiosensitive structure that is often unavoidably included in standard RT fields. RT can result in lasting pain, functional impairment, and decreased QOL, even at standard fractionation doses. As high-dose RT for oligometastatic disease becomes more standard in current practice, and as cancer-specific survival continues to improve, we can only expect an increase in the incidence of RT-induced bone toxicity. Preclinical animal models including murine and NHP studies have been crucial to our understanding of how irradiation affects the complex processes governing the bone microenvironment. Furthermore, these animal models have been instrumental to the creation and implementation of new translational clinical trials with therapeutic targets.
